# Perineural Spread of Salivary Duct Carcinoma to the Internal Auditory Canal

**DOI:** 10.1155/2014/476317

**Published:** 2014-11-26

**Authors:** Winfred Kitavi, Ulrich Hamberger, Holger Sudhoff

**Affiliations:** ^1^Department of Otorhinolaryngology, Head and Neck Surgery, Klinikum Bielefeld, Münster University, Teutoburger Straße 50, 33604 Bielefeld, Germany; ^2^Department of Pathology, Klinikum Bielefeld, 33604 Bielefeld, Germany

## Abstract

Salivary duct carcinomas (SDCs) are high-grade malignant tumors exhibiting aggressive growth with early regional and distant metastasis. We report a case of SDC in a 63-year-old male with early recurrent disease in the cerebellopontine angle (CPA) after total parotidectomy and adjuvant radiotherapy. The tendency of the tumor to recur or metastasize despite radical surgical measures and radiotherapy continues to pose a therapeutic challenge.

## 1. Introduction

Salivary duct carcinoma is a rare malignant epithelial tumor that predominantly occurs in the parotid gland [1, 2]. It represents 1%–3% of salivary gland neoplasms and is most common amongst male patients in the 6th or 7th decade of life. The tumor exhibits a histopathological similarity to ductal carcinoma of the female breast and has an aggressive clinical course with a mean survival of three years following primary diagnosis [3–6]. Approximately 250 cases of salivary duct carcinoma have been reported worldwide [7]. We report the case of a 63-year-old patient who presented with right-sided facial nerve palsy of insidious onset and a mass in the right parotid gland. In spite of radical surgical approach and adjuvant radiotherapy, the patient presented with dizziness only 8 months after treatment with MRI revealing a lesion in the internal auditory canal, thus displaying the propensity of this tumor to metastasize.

## 2. Case Presentation

**Figure 1:**
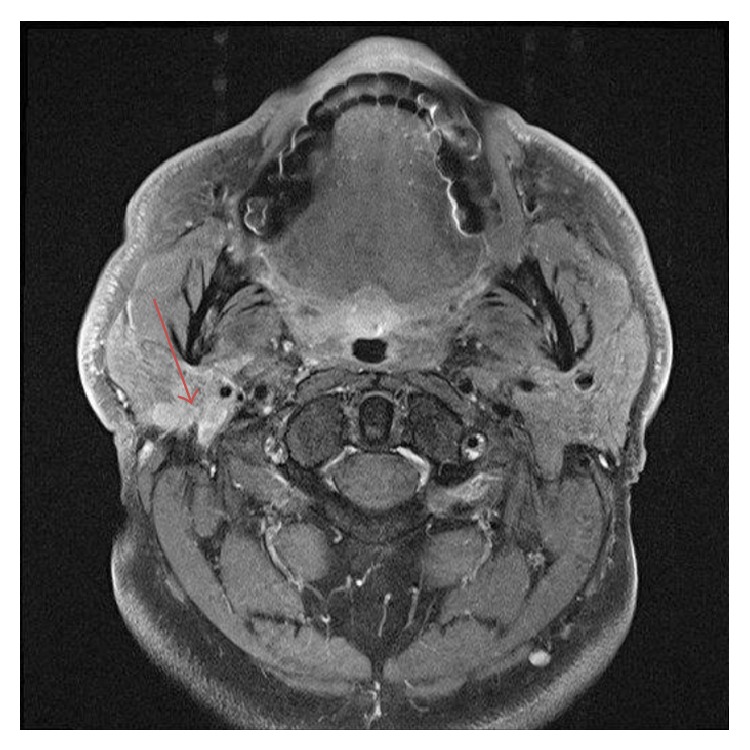
MRI of the neck, axial section (T1-weighted), showing a mass in the deep lobe of the right parotid gland (arrow).

A 63-year-old male presented with a complete facial nerve palsy of the right side of insidious onset a year prior to presentation. Physical examination revealed a mass in the right parotid gland (Figure 1). There was no cervical lymphadenopathy. Except for well-controlled hypertension, the patient was otherwise in good health and had no history of malignant disease. MRI of the neck showed a hyperintense lesion involving the deep lobe of the parotid gland. A right total parotidectomy with excision of the facial nerve and neck dissection was performed. In the same procedure a static reconstruction of the defect with static suspension, the lower face with fascia lata, and lower lid tightening were undertaken.

**Figure 2:**
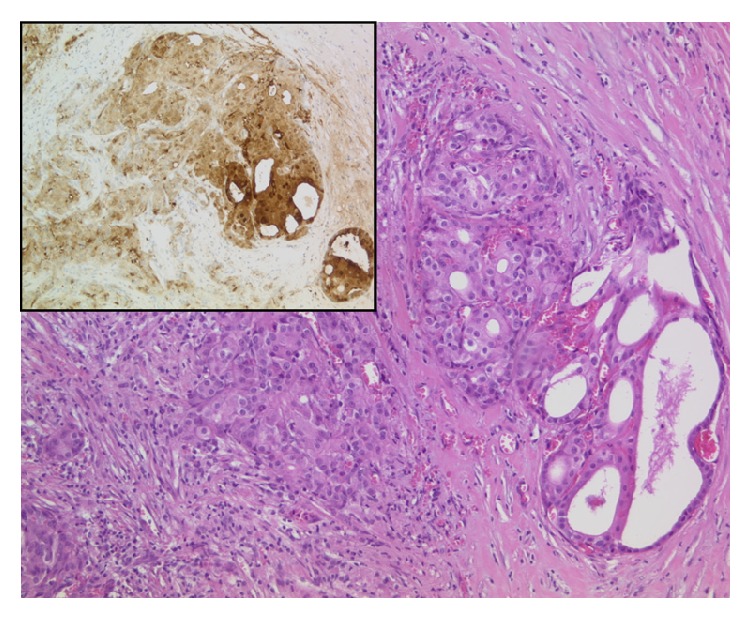
Postoperative histopathologic examinations showing the salivary duct carcinoma (×100). Inset: GCDFP 15-immunohistochemistry (positive in invasive and intraductal salivary duct carcinoma (×20)).

The surgical specimen showed an unencapsulated and poorly circumscribed multinodulary tumor. Histopathological examination of the specimen revealed a carcinoma with island and nests of moderate to marked pleomorphic neoplastic cells. An intraductal tumor component and perineural invasion and vascular invasion were described (Figure 2). Immunohistochemistry showed cytokeratin 7, GCDFP15 (BRST-2), and EMA positivity as well as elevated Ki-67 expression confirming a high-grade salivary duct carcinoma of the parotid gland.

**Figure 3:**
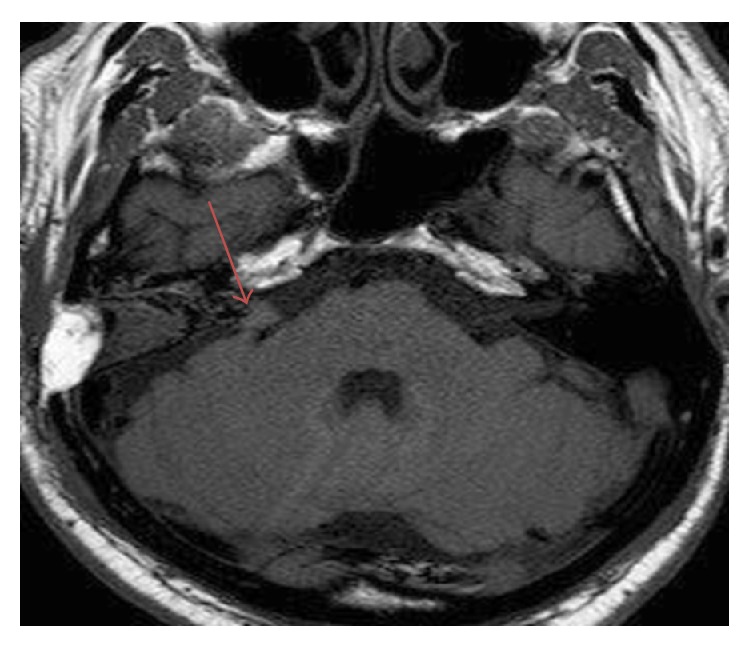
MRI-scan (axial section) demonstrating a contrast enhancing lesion in the right internal auditory canal.

A chest X-ray, abdominal ultrasound, and a gadolinium-enhanced brain MRI were performed to rule out distant metastases. The patient was discharged in a stable condition and adjuvant radiotherapy preformed. Eight months after treatment the patient presented to the department with vertigo and progressive right-sided hearing loss. Magnetic resonance imaging showed a contrast enhancing lesion in the right internal auditory canal resembling a vestibular schwannoma of the cerebellopontine angle (CPA) (Figure 3). Owing to the medical history, involvement of the intracranial portion of the facial nerve was presumed.

Following CT-scans to exclude further lesions, surgical removal was undertaken via translabyrinthine approach and clear margins at the entry zone of the facial nerve at the brainstem were obtained. Histopathological examination confirmed our presumption. The patient recovered quickly and was discharged in relatively stable condition. Six months after revision-surgery the patient was in a stable condition and has had no further progression of the disease.

## 3. Discussion

Salivary duct carcinoma was first described by Kleinsasser et al. in 1968 but it was not until 1991 that the World Health Organisation (WHO) formally recognized this tumor entity as a classification of salivary gland tumor [1, 2]. It is a high-grade neoplasm of the salivary glands mostly affecting the parotid gland (87%) with only very few cases involving the submandibular and minor salivary glands. It accounts for 1%–3% of all malignant tumors of the salivary gland and has a male predominance. 75% of the patients are male aged between 60 and 70 years [7].

This tumor has invasive growth with early lymphovascular and perineural invasion. Local recurrence and distant metastases to the lung liver and bone are frequent leading to a poor prognosis. Distant metastases are the most common cause of tumor-associated morbidity. Clinical presentation may occur as lesions in the parotid region, facial nerve dysfunction, cervical lymphadenopathy, facial nerve palsy, and facial pain [8].

Salivary duct carcinoma can be established from pleomorphic adenoma (carcinoma ex pleomorphic adenoma) or de novo. It has also been reported to arise from longstanding chronic obstructive sialadenitis [9]. Differential diagnosis includes metastatic breast carcinoma, cystadenocarcinoma, polymorphous low-grade adenocarcinoma, and oncocytic carcinoma. There is no consensus regarding the treatment of this tumor [10]. Aggressive multimodality appears to be the appropriate treatment for salivary duct carcinoma. The majority of reported cases were treated with radical surgical approach and adjuvant radiotherapy. Neck dissection should always be performed to reduce the risk of recurrence [10].

## 4. Conclusion

Salivary duct carcinoma represents a highly aggressive tumor. The tendency of the tumor to recur and metastasize despite radical surgical measures and adjuvant radiotherapy continues to pose a therapeutic challenge.
